# Relationship between Community or Home Gardening and Health of the Elderly: A Web-Based Cross-Sectional Survey in Japan

**DOI:** 10.3390/ijerph16081389

**Published:** 2019-04-17

**Authors:** Daisuke Machida

**Affiliations:** Department of Health and Nutrition, Takasaki University of Health and Welfare, Takasaki, Gunma 370-0033, Japan; machida-d@takasaki-u.ac.jp; Tel.: +81-27-352-1290

**Keywords:** gardening, community garden, home garden, health, lifestyle, subjective happiness, physical activity, vegetable intake, social cohesion, Japan

## Abstract

There have been many reports indicating the relationship between gardening and health or healthy lifestyles among adults in developed countries all over the world. However, Japanese evidence is lacking. The aim of this study was to clarify the relationship between community or home gardening and health status or a healthy lifestyle using a web-based survey with Japanese elderly living in the community. A survey was conducted to gather data from 500 gardeners and 500 nongardeners aged 60 to 69. As a result, significant relationships were shown between community gardening and exercise habits, physical activity, eating vegetables, and connections with neighbors. Moreover, the significant relationships between home gardening and the following items were indicated: Subjective happiness, exercise habits, physical activity, sitting time, eating breakfast, eating vegetables, eating balanced meals, and connections with neighbors. No item demonstrated a significant relationship with gardening frequency. A significant relationship was demonstrated between gardening duration and health problems affecting everyday life. Further significant relationships were shown between gardening with others and subjective happiness, having a reason for living. In conclusion, promising positive relationships between community or home gardening and health or healthy lifestyles were indicated.

## 1. Introduction

There have been many reports that have indicated a positive relationship between gardening and health or healthy lifestyles among adults in developed countries all over the world [1,2,3,4,5,6,7,8]. They have reported the positive effects as follows: Greater fruit and vegetable intake [1,2,3,4,5,6,7], physical activity [1,3,4,5,8], self-rated health [5], body mass index (BMI) [3,5,8], mental health [1,4,5,6,8], and social well-being [1,4,5,6,8], not only among patients but also the wider community. In previous studies, the effects of different types of gardening have been examined, including urban gardening [1,2], community gardening [3,4,5,7], and allotment gardening [6]. An allotment garden is a garden divided into parcels and leased to participants [6], whereas a community garden is essentially a common garden used by community members but in a broader sense includes allotment gardens as well in North America [3,5,6]. These gardens can be seen in both urban and rural settings and in both developing and developed countries [3,5].

Japan’s aging rate is one of the highest globally [9]; the fact that gardening can help in improving the health of the elderly in Japan is an important finding from a global perspective. However, in Japan, there have only been two studies on gardening and health or healthy lifestyles in the general community [10,11]. One study was conducted in Tokyo, the capital of Japan and one of the most urbanized areas in the world [10]. In this study, the relationship between community gardening and health was analyzed [10]. Positive relationships between community gardening and mental health, subjective health, social cohesiveness, and vegetable intake frequency were reported [10]. Another study was conducted in a city in Gunma Prefecture, which is a suburban setting [11]. This study analyzed the relationship between home or community gardening and health or healthy lifestyles [11]. However, the study was limited by a small sample size [11]. Moreover, some of the results of these two studies were inconsistent [10,11]. For example, in the suburban area, there was no significant association between fruit and vegetable intake frequency or subjective health and community gardening [11]. Therefore, it is necessary to accumulate further evidence in Japan about these topics.

The aim of this study was to clarify the relationship between community or home gardening and health status or a healthy lifestyle using a web-based survey for Japanese elderly living in the community. After comparing findings with those from previous studies, the relationship between gardening and health in Japan is discussed.

## 2. Materials and Methods

### 2.1. Study Design and Participants

This was a web-based cross-sectional study in Japan. The survey was conducted by Cross Marketing Inc. [12] on 6 December 2012, as a part of the Committee on Evidence on Farm Work and Health by the Japanese Ministry of Agriculture, Forestry and Fisheries [13]. Cross Marketing Inc. is a survey company with approximately 4.2 million people registered, the largest in Japan, and it is also possible to specify the characteristics of the surveyed population [12]. Here the survey was conducted to gather data on 500 gardeners and 500 nongardeners aged 60–69 [13]. In addition, professional farmers were excluded from the participants [13]. Moreover, respondents included were from all 47 prefectures (administrative subdivision) in Japan [13]. To prevent only those who were concerned about agricultural work and health from responding to the survey, the terms “farm work” and “health” were omitted from the research title, which was entitled “Questionnaire Survey on Daily Life” [13].

There were no ethical problems conducting this study. All participants provided informed consent for inclusion before they participated in the study. The survey was conducted anonymously, and all responses were optional [13]. When conducting this research, no issue arose that contradicted the Declaration of Helsinki. The survey data were published on the website of the Ministry of Agriculture, Forestry, and Fisheries [13]. The data were used after obtaining permission from the person in charge of the Ministry of Agriculture, Forestry, and Fisheries. What are disclosed are anonymous data, and outside the scope of the ethical guidelines concerning medical research on human subjects in Japan [14]. Therefore, review and approval from the Takasaki University of Health and Welfare, IRB office, was not requested.

### 2.2. Outcomes: Health Status and Healthy Lifestyle

To assess health status, items were used as follows: Subjective symptoms, periodic visit with illness or injury to clinic, health problems affecting everyday life, subjective happiness, feeling a reason for living, psychological distress, and BMI.

Subjective symptoms, periodic visits with illness or injury to clinic, and health problems affecting everyday life were indicated as “present (yes)” or “none”. These three items were used in Comprehensive Survey of Living Conditions in Japan [15].

Subjective happiness was rated on an 11-point scale (unhappy = 0 to happy = 10). This item has been used in previous research studies [16,17]. The reliability of measuring happiness levels using a single item has been clarified by a previous study [18]. On the basis of previous reports [16,17], we divided the participants into two groups using the median happiness of the Japanese, that is, less than 6 and 7 or more.

The participants were asked “Do you feel a reason to live (fun and pleasure)?” and rated as “none, little, moderate, a lot”. This was the item used by an investigation of the Cabinet Office in Japan [19]. The answers were divided as “little or none” and “moderate or a lot”.

Psychological distress was rated using four items among six items of the K6 [20], Japanese version [21] (nervous, restless or fidgety, so sad that nothing could cheer you up, and everything was an effort; questions about hopelessness and worthlessness were not asked). The total score ranged from 0 to 16. Moreover, Cronbach’s alpha for the four items was 0.850, and there was no problem in internal reliability. In the previous studies using K6, less than 12 points was regarded as a cutoff point [22,23]. Since only four items were used this time, participants were classified into two groups according to their K6 score of less than 9 points.

For BMI, they were asked their height and weight and it was calculated from there. On the basis of the BMI standard value for people aged 60 years or older according to Dietary Reference Intakes for the Japanese (2015) [24], they were divided into three groups: Underweight (<20), normal weight (20–24.9), and overweight or obese (≥24.9).

To assess a healthy lifestyle, the following items were used: Exercise habits, physical activity, sitting time, walking speed, eating breakfast, eating vegetables, eating balanced meals, rest due to sleep, connections with neighbors, and having friends.

Exercise habits referred to exercising continuously for more than 30 min, for more than 2 days a week, for more than 1 year. Physical activity referred to whether walking or physical activity equal to or more than daily life activity was carried out for more than 1 h a day. These are items from the standard questionnaire used for specific health check-up in Japan [25].

Sitting time referred to “How long you spend sitting per day while you are awake normally?”, and answers were obtained for “less than 3 hours”, “3 to 6 hours”, and “6 hours or more”. It has been reported that if sitting time is extensive, the risk of mortality due to total cause cardiovascular disease is high [26,27].

The walking speed referred to “If the speed of walking was faster than that of the same generation of the same gender?”, and answers were obtained with “yes” or “no”. This item is from a specific health check-up questionnaire used in Japan [25].

Breakfast intake referred to “Do you eat breakfast usually?” and answers included the following options: “I eat it every day”, “I do not eat it 2–3 days a week”, “I do not eat it 4–5 days a week”, and “almost do not eat it”. This item was used in the National Health and Nutrition Survey of the Ministry of Health, Labor, and Welfare [28]. On the basis of national recommendations [29], it was divided into “eat every day” and “not eating every day”.

Vegetable consumption was assessed as follows: “Usually, do you eat enough vegetables?” and answered “enough”, “moderate”, “not enough”, and “shortage”. It was divided into “enough or moderate” and “not enough or shortage.” Increased vegetable intake reduces the risk of total mortality and mortality due to cardiovascular disease [30,31].

Eating balanced meals was assessed as follows: “How many days do you eat meals consisting of grain, fish and meat, and vegetable dishes, two or more times/day?” and the answers were “almost every day”, “4 to 5 days/week”, “2 to 3 days/week”, and “almost none”. This was the item used in the investigation of the Cabinet Office in Japan [32]. On the basis of national recommendations [33], it was divided into “eat every day” and “not eating every day”.

Rest due to sleep was assessed as follows: “Over the past month, are you well rested by sleeping?” and answered “enough”, “moderate”, “not enough”, and “shortage”. It was divided into “enough or moderate” and “not enough or shortage”. This item was used in the Comprehensive Survey of Living Conditions in Japan [15].

Connections with neighbors were assessed by asking, “Do you think that the connection between you and your neighbors is strong?” and answered “strong”, “somewhat strong”, “somewhat weak”, “weak”, and “unknown”. It was divided into “strong” and “weak or unknown”. Having friends was assessed by asking “How many close friends do you have?”, and answers available were “a lot”, “moderate”, “little”, and “none”. It was divided into “a lot or moderate” and “little or none”. Both of these items were used in the investigation of the Cabinet Office in Japan [19,34].

### 2.3. Gardening Style and Status

The participants were asked questions regarding their gardening styles. “Do you do farm work? Please answer at least twice a month and not a few times in a year”, and the participants answered as “Working at a farm is my job”, “I do farm work in community garden as a hobby or leisure activity”, “I do farm work in my home garden, which has an area of 15 square meters, as a hobby or leisure activity”, and “I do not do farm work”. Those who answered “Working at a farm is my job” were not included into the data set. The responses were treated as three groups in the analyses: Community gardener, home gardener, and nongardener.

The frequency of gardening status was also assessed by asking “How many days have you been gardening each week in the past a month?” Answers were in the range of 0 to 7 (day/week). The duration of gardening time of each day was requested, and the duration per week was calculated by combining the frequency and gardening time each day (hours/week). Moreover, to ascertain whether gardening took place alone or with others, the questionnaire asked: “Who do you do garden with?” Responses included “almost alone” or “often with friends or family”.

### 2.4. Analysis

First, the relationship between community or home gardening with health status and healthy lifestyles (*N* = 1000) was analyzed. One-way ANOVA for age, *t*-test for gardening frequency and duration, and χ^2^ test for all other items were conducted. If there was a significant difference, a post hoc test using Bonferroni correction was conducted. Then, unadjusted and adjusted odds ratios using gardening styles as independent variables were calculated (Ref. nongardener). Multinomial logistic regression models were used for BMI and sitting time, and binary logistic regression models were used for all other items. Sex, age, family structure, and employment status were used as covariables in adjusted models. Additionally, among gardeners, the relationship between gardening status and health status or healthy lifestyles (*N* = 500) was analyzed. Gardening status was used as an independent variable, and adjusted odds ratios were calculated. Gardening style was added for an adjusted model and analyzed similarly with that described above, according to outcomes.

All statistical tests described here were two-sided and performed using IBM SPSS Statistics version 23 (IBM, Armonk, NY, USA). Differences of *p* < 0.05 were accepted as significant.

## 3. Results

### 3.1. Sample Characteristics

The distribution of the responses of 1000 participants according to gardening style is presented in Table 1. Basic characteristics were significantly different in sex, age, family structure, and employment status. In post hoc tests, there was a greater population of women, and living alone among the home gardener group, and of workers among the community gardener group, than the nongardener group. Home gardeners and community gardeners were older than nongardeners. Therefore, it was reasonable to adjust these variables in adjusted models. For the gardening status, frequency, duration, and gardening with others were similar between home gardeners and community gardeners. For outcomes, there were significant differences in subjective happiness, reasons for living, exercise habit, physical activity, sitting time, eating breakfast, eating vegetables, eating balanced meals, connection with neighbors, and having friends. In post hoc tests, compared with the nongardener group, it was discovered that a more significant population had feelings of happiness and reasons for living, had exercise and physical activity habits, sat for short times, ate breakfast and a balanced meal every day, consumed enough vegetables, and had strong connections with neighbors among the home gardener group; and exercise and physical activity habits, ate enough vegetables, had stronger connections with neighbors, and had relatively many friends among the community gardener group. 

### 3.2. Gardening and Health Status or Healthy Lifestyle

The results of the analysis revealing the relationship between community or home gardening and health status or healthy lifestyle are shown in Table 2. Significant relationships were shown in the adjusted model between community gardening and exercise habits, physical activity, eating vegetables, and connections with neighbors. Moreover, significant relationships between home gardening and the following items were shown: Subjective happiness, exercise habits, physical activity, sitting time, eating breakfast, eating vegetables, eating balanced meals, and connections with neighbors. All of these showed that gardening was positively related to health and a healthy lifestyle. The significant relationships between community or home gardening and feeling a reason for living, or having friends, were shown in unadjusted models. However, the results of adjusted models also revealed these trends, but they were not significant. Similarly, significant relationships between community gardening and sitting time, walking speed, and eating balanced meals were shown in unadjusted models, but these trends were not significant in adjusted models.

### 3.3. Gardening Status and Health Status or Healthy Lifestyle

Results analyzing the relationship between gardening status and health status or healthy lifestyle are shown in Table 3. There were no items with a significant relationship to gardening frequency. A significant relationship was shown between gardening duration and health problems affecting everyday life. Significant relationships were shown between gardening with others and subjective happiness, feeling a reason for living, and sitting time. Subjective happiness and feeling a reason for living were positively correlated with health and with gardening with others. On the other hand, sitting time was negatively correlated with health. That is, people who garden with others tend to spend a longer time sitting.

## 4. Discussion

This study clarified the relationship between community or home gardening and health status or a healthy lifestyle for Japanese elderly living in the community. The results reveal the positive relationship between community gardening and exercise habits, physical activity, eating vegetables, and connections with neighbors; and between home gardening and subjective happiness, exercise habits, physical activity, sitting time, eating breakfast, eating vegetables, eating balanced meals, and connections with neighbors. Overall, gardening was positively associated with health and healthy lifestyles among the elderly in Japan. In other words, it was suggested that gardening contributes to promoting health for the elderly in Japan. Moreover, positive relationships between the time spent gardening and health problems affecting everyday life, between gardening with others and subjective happiness, and feeling a reason for living were shown. However, there was a negative relationship between gardening with others and sitting time. The presence or absence of such differences because of gardening status provides useful suggestions for implementing health promotion by gardening.

The relationships between exercise habits, physical activity, eating vegetables, and connections with neighbors were common to both community gardening and home gardening. This study was the first to confirm the relationship between gardening and exercise habits. Those who are gardening are active, and it seems that they are exercising on a daily basis. In addition, these findings on the relationship between gardening and physical activity are not consistent with the previous studies conducted in Japan [10,11]. There was no difference in an urban study [10]. On the other hand, a study in a suburban area indicated that gardeners tend to be very physically active [11]. When considering results over time, gardeners tend to be more physically active as a whole. Even in a study in the Netherlands, it was reported that allotment gardeners were much more physically active than their neighbors over the summer [35]. Generally, in Japan, many people choose walking as transportation in urban areas and tend to be quite active. On the other hand, in suburban areas, many more people use cars and are less physically active. There are perhaps many people with high levels of physical activity level in urban areas, where gardening does not contribute much to physical activity. However, in suburban areas, gardening provides a good opportunity for physical activity. Moreover, positive associations between gardening and vegetable intake have also been confirmed in previous studies in Japan [10,11] and the United States [36,37,38,39,40]. Therefore, the relationship between gardening and a large vegetable intake is definite. Furthermore, the association between gardening and social cohesion has been confirmed in prior Japanese research [10,11]. Similarly, the relationship between gardening and social involvement or perceived collective efficacy have also been reported in the United States [41]. By increasing opportunities to go out, contacts with the local residents become more frequent, and connections become stronger.

The relationships between subjective happiness, sitting time, eating breakfast, and eating balanced meals were different depending on whether community gardening or home gardening was involved. Sitting time odds ratios were similar for the community gardener and home gardener. Thus, there was a possible β error due to a small sample of community gardeners. Because of the similar trends observed, differences were not associated with gardening style. In a previous study in Japan, a similar sitting time for all kinds of gardeners was also reported [11]. In addition, the relationship between subjective happiness, eating breakfast, and eating balanced meals tended to be different between home gardeners and community gardeners. For balanced meals, studies in the United States have identified positive associations with community gardening [7]. As a hypothesis, it could be inferred that there were conflicts with economic status, because the relationship between these items and economic status has been reported in Japan [17,42,43,44,45]. People who have a home garden may be economically better off. In this survey, economic status was not assessed, so it will be necessary to examine correlations with economic status in the future.

It was suggested that a mutual relationship exists between gardening duration and health problems affecting everyday life, such that, for those without a health problem, long-term gardening is possible, and gardening keeps them healthy. There were no other items related to the frequency or duration of gardening. This result is consistent with a previous study [10], which found that irrespective of frequency and duration, gardening is related to the health and healthy lifestyles recognized in this study. A new implication is that people working alongside others feel a reason for living and more happiness. Gardening with others is a part of social participation and may increase the gardener’s happiness and pleasure [40,46]. Therefore, it would be good to do gardening with others. For example, gardening with family members, or having opportunities to gather in harvest festivals at community gardens to promote gardening with other people, may lead to a feeling of happiness and reason for living. However, there are not only positive outcomes to shared gardening. According to the results of this study, those who do gardening with others also spend a longer time sitting.

### Limitations

These were self-reported data and have a probability of recall bias [47]. The gardeners who believe that gardening is good for health may systematically overestimate their own health status and healthy lifestyle, as health benefits related to gardening are widely known [1,2,3,4,5,6,7,8]. Additionally, this research was an Internet survey, and the possibility of sampling bias cannot be denied. Further, participants were only aged 60 to 69 years, and it is not known if the same trend exists in other age groups. Even those targeting adults in Japan in previous research were about 60 years old on average [10,11], and a question to consider in the future is whether gardening will be as effective for younger adults in Japan. Finally, this was a cross-sectional study, and further longitudinal studies are required to clarify the causal relationships.

## 5. Conclusions

This study examined the relationships between community or home gardening and health or healthy lifestyles for Japanese people aged 60–69 living in the community. The results revealed promising relationships between health and gardening. Moreover, many reports have indicated a positive relationship between gardening and health or healthy lifestyles in adults in developed countries all over the world [1,2,3,4,5,6,7,8]. According to the above, gardening contributes positively to the health of Japanese people aged 60 years and older living in the community. It is hoped that the promotion of health through gardening will be practiced more often in Japan.

## Figures and Tables

**Table 1:** Distribution of responses.

Outcomes	Total		Community Gardener (C)		Home Gardener (H)		Nongardener (N)		*p*-Value * (Post Hoc Test) **
	*n* (Mean)	% (SD)	*n* (Mean)	% (SD)	*n* (Mean)	% (SD)	*n* (Mean)	% (SD)	
**Basic characteristics**									
sex									
men	694	69.4	87	67.4	280	75.5	327	65.4	0.005
women	306	30.6	42	32.6	91	24.5	173	34.6	(women: N > H)
age	(63.6)	(2.6)	(64.1)	(2.6)	(63.9)	(2.7)	(63.3)	(2.5)	0.001 (CH > N)
family structure									
living with two or more members	901	90.1	119	92.2	351	94.6	431	86.2	<0.001
living alone	99	9.9	10	7.8	20	5.4	69	13.8	(living alone: N > H)
employment status									
unemployed or retired	527	52.7	57	44.2	212	57.1	258	51.6	0.031
working at least a day/week	473	47.3	72	55.8	159	42.9	242	48.4	(working: C > N)
**Gardening status**									
frequency (days/week)	(2.3)	(1.5)	(2.3)	(1.4)	(2.3)	(1.5)	―	―	0.933
duration (hour/week)	(4.2)	(4.2)	(3.9)	(3.5)	(4.2)	(4.4)	―	―	0.449
gardening with others									
almost alone	324	32.4	76	58.9	248	66.8	―	―	0.104
often with friends or family	176	17.6	53	41.1	123	33.2	―	―	
**Health status**									
subjective symptoms									
present	292	29.2	29	22.5	115	31.0	148	29.6	0.179
none	708	70.8	100	77.5	256	69.0	352	70.4	
periodic visit with illness or injury to clinic									
yes	485	48.5	54	41.9	193	52.0	238	47.6	0.118
none	515	51.5	75	58.1	178	48.0	262	52.4	
health problems affecting everyday life									
present	121	12.1	11	8.5	49	13.2	61	12.2	0.371
none	879	87.9	118	91.5	322	86.8	439	87.8	
subjective happiness (on a scale from 0 to 10)									
less than 6	322	32.2	48	37.2	93	25.1	181	36.2	0.001
7 or more	678	67.8	81	62.8	278	74.9	319	63.8	(7 or more: H > CN)
reasons for living									
little or none	182	18.2	17	13.2	56	15.1	109	21.8	0.011
moderate or a lot	818	81.8	112	86.8	315	84.9	391	78.2	(moderate or a lot: H > N)
psychological distress									
low (less than 9)	866	86.6	116	89.9	325	87.6	425	85.0	0.266
high (9 or more)	134	13.4	13	10.1	46	12.4	75	15.0	
body mass index (kg/m^2^)									
underweight (<20)	148	14.8	18	14.0	50	13.5	80	16.0	0.246
normal weight (20–24.9)	646	64.6	92	71.3	235	63.3	319	63.8	
overweight or obese (≥25)	206	20.6	19	14.7	86	23.2	101	20.2	
**Healthy lifestyles**									
exercise habit (continuously for more than 30 minutes, for more than 2 days a week, for more than 1 year)									
no	531	53.1	57	44.2	175	47.2	299	59.8	<0.001
yes	469	46.9	72	55.8	196	52.8	201	40.2	(yes: CH > N)
physical activity (more than 1 hour/day)									
no	380	38.0	34	26.4	114	30.7	232	46.4	<0.001
yes	620	62.0	95	73.6	257	69.3	268	53.6	(yes: CH > N)
sitting time									
≥6 hours/day	272	27.2	29	22.5	81	21.8	162	32.4	0.005 (<3 hours/day: H > N)
3–6 hours/day	506	50.6	66	51.2	200	53.9	240	48.0	
<3 hours/day	222	22.2	34	26.4	90	24.3	98	19.6	
walking speed (If the speed of walking was faster than that of the same generation of the same gender?)									
no	351	35.1	37	28.7	123	33.2	191	38.2	0.080
yes	649	64.9	92	71.3	248	66.8	309	61.8	
eating breakfast									
not every day	77	7.7	10	7.8	18	4.9	49	9.8	0.026
eat every day	923	92.3	119	92.2	353	95.1	451	90.2	(eat every day: H > N)
eating vegetables									
not enough or shortage	727	72.7	88	68.2	236	63.6	403	80.6	<0.001
enough or moderate	273	27.3	41	31.8	135	36.4	97	19.4	(enough or moderate: CH > N)
eating balanced meals (2 or more times/day)									
not every day	336	33.6	38	29.5	95	25.6	203	40.6	<0.001
eat every day	664	66.4	91	70.5	276	74.4	297	59.4	(eat every day: H > N)
rest due to sleep									
not enough or shortage	150	15.0	18	14.0	53	14.3	79	15.8	0.775
enough or moderate	850	85.0	111	86.0	318	85.7	421	84.2	
connection with neighbors									
weak or unknown	700	70.0	80	62.0	229	61.7	391	78.2	<0.001
strong	300	30.0	49	38.0	142	38.3	109	21.8	(strong: CH > N)
having friends									
little or none	467	46.7	50	38.8	161	43.4	256	51.2	0.011
moderate or a lot	533	53.3	79	61.2	210	56.6	244	48.8	(moderate or a lot: C > N)
*N*	1000	100	129	100	371	100	500	100	

SD: Standard deviation. * Age: One-way ANOVA, frequency and duration: *t*-test, other items: χ^2^ test. ** Bonferroni correction, > means significant difference of <0.05 level.

**Table 2:** Relationship between gardening and health status or healthy lifestyles.

Outcomes	Unadjusted		Adjusted	
	Community Gardener	Home Gardener	Community Gardener	Home Gardener
	OR (95%CI)	OR (95%CI)	OR (95%CI)	OR (95%CI)
**Health status**				
subjective symptoms (Ref. present)				
none	1.45 (0.92–2.29)	0.94 (0.70–1.25)	1.54 (0.97–2.45)	0.98 (0.73–1.32)
periodic visit with illness or injury to clinic (Ref. yes)				
none	1.26 (0.85–1.87)	0.84 (0.64–1.10)	1.31 (0.88–1.95)	0.90 (0.68–1.18)
health problems affecting everyday life (Ref. present)				
none	1.49 (0.76–2.92)	0.91 (0.61–1.37)	1.58 (0.80–3.12)	0.99 (0.66–1.49)
subjective happiness (on a scale from 0 to 10; Ref. less than 6)				
7 or more	0.96 (0.64–1.43)	**1.70 (1.26–2.28)**	0.89 (0.59–1.34)	**1.60 (1.18–2.16)**
reasons for living (Ref. little or none)				
moderate or a lot	**1.84 (1.06–3.19)**	**1.57 (1.10–2.24)**	1.58 (0.90–2.77)	1.40 (0.97–2.02)
psychological distress (Ref. low (less than 9))				
high (9 or more)	0.64 (0.34–1.18)	0.80 (0.54–1.19)	0.72 (0.38–1.36)	0.85 (0.57–1.27)
body mass index (kg/m^2^; Ref. normal weight (20–24.9))				
underweight (<20)	0.78 (0.44–1.37)	0.85 (0.57–1.25)	0.83 (0.46–1.48)	0.97 (0.65–1.46)
overweight or obese (≥25)	0.65 (0.38–1.12)	1.16 (0.83–1.61)	0.69 (0.40–1.19)	1.10 (0.78–1.55)
**Healthy lifestyles**				
exercise habit (continuously for more than 30 minutes, for more than 2 days a week, for more than 1 year; Ref. no)				
yes	**1.88 (1.27–2.78)**	**1.67 (1.27–2.18)**	**1.79 (1.20–2.67)**	**1.57 (1.19–2.07)**
physical activity (more than 1 hour/day; Ref. no)				
yes	**2.42 (1.57–3.72)**	**1.95 (1.47–2.59)**	**2.32 (1.50–3.59)**	**1.94 (1.45–2.59)**
sitting time (ref. ≥6 hours/day)				
3~6 hours/day	**1.54 (0.95–2.48)**	**1.67 (1.20–2.31)**	1.47 (0.91–2.39)	**1.59 (1.14–2.22)**
<3 hours/day	**1.94 (1.11–3.38)**	**1.84 (1.24–2.72)**	1.74 (0.99–3.05)	**1.80 (1.21–2.69)**
walking speed (If the speed of walking was faster than that of the same generation of the same gender; Ref. no)				
yes	**1.54 (1.01–2.34)**	1.25 (0.94–1.65)	1.48 (0.96–2.26)	1.22 (0.92–1.63)
eating breakfast (Ref. not every day)				
eat every day	1.29 (0.64–2.63)	**2.13 (1.22–3.72)**	1.21 (0.59–2.48)	**1.94 (1.10–3.43)**
eating vegetables (Ref. not enough or shortage)				
enough or moderate	**1.94 (1.26–2.98)**	**2.38 (1.75–3.23)**	**1.83 (1.18–2.85)**	**2.29 (1.67–3.14)**
eating balanced meals (2 or more times/day; Ref. not every day)				
eat every day	**1.64 (1.08–2.49)**	**1.99 (1.48–2.66)**	1.48 (0.97–2.27)	**1.80 (1.33–2.44)**
rest due to sleep (Ref. not enough or shortage)				
enough or moderate	1.16 (0.67–2.01)	1.13 (0.77–1.64)	1.11 (0.63–1.96)	0.99 (0.67–1.46)
connection with neighbors (Ref. weak or unknown)				
strong	**2.20 (1.45–3.32)**	**2.22 (1.65–3.00)**	**2.03 (1.33–3.09)**	**2.08 (1.53–2.82)**
having friends (Ref. little or none)				
moderate or a lot	**1.66 (1.12–2.46)**	**1.37 (1.04–1.79)**	1.49 (0.99–2.24)	1.28 (0.97–1.70)

*N* = 1000 (community gardener: *n* = 129, home gardener: *n* = 371, nongardener (Ref.): *n* = 500). *Adjusted for sex, age, family structure, and employment status. OR: Odds ratio, CI: Confidence interval, **Bold** means statistically significant (*p* < 0.05).

**Table 3:** Relationship between gardening status and health status or healthy lifestyle.

Outcomes	Gardening Status		
	Frequency	Duration	Gardening with Others
	(Day/Week)	(Hour/Week)	(Ref. Almost Alone)
	OR (95%CI)	OR (95%CI)	OR (95%CI)
**Health status**			
subjective symptoms (Ref. present)			
none	1.06 (0.87–1.29)	1.02 (0.95–1.10)	1.53 (0.99–2.37)
periodic visit with illness or injury to clinic (Ref. yes)			
none	0.97 (0.81–1.14)	1.00 (0.94–1.06)	1.03 (0.70–1.51)
health problems affecting everyday life (Ref. present)			
none	0.87 (0.68–1.13)	**1.13 (1.00–1.27)**	1.25 (0.69–2.26)
subjective happiness (on a scale from 0 to 10; Ref. less than 6)			
7 or more	0.94 (0.78–1.14)	1.03 (0.96–1.10)	**2.14 (1.34–3.41)**
reasons for living (Ref. little or none)			
moderate or a lot	0.98 (0.77–1.25)	1.03 (0.94–1.13)	**2.02 (1.10–3.74)**
psychological distress (Ref. low (less than 9))			
high (9 or more)	0.97 (0.74–1.27)	1.01 (0.92–1.11)	0.72 (0.38–1.35)
body mass index (kg/m^2^; Ref. normal weight (20–24.9))			
underweight (<20)	0.91 (0.74–1.11)	1.01 (0.92–1.12)	0.63 (0.35–1.15)
overweight or obese (≥25)	0.92 (0.79–1.08)	1.01 (0.93–1.08)	0.98 (0.61–1.60)
**Healthy lifestyles**			
exercise habit (continuously for more than 30 minutes, for more than 2 days a week, for more than 1 year; Ref. no)			
yes	1.10 (0.93–1.31)	0.96 (0.91–1.02)	1.20 (0.81–1.78)
physical activity (more than 1 hour/day; Ref. no)			
yes	1.18 (0.96–1.45)	1.02 (0.95–1.10)	0.87 (0.57–1.34)
sitting time (ref. ≥6 hours/day)			
3~6 hours/day	0.84 (0.68–1.04)	1.01 (0.94–1.09)	**0.52 (0.32–0.84)**
<3 hours/day	0.95 (0.75–1.21)	1.02 (0.93–1.10)	**0.55 (0.31–0.96)**
walking speed (If the speed of walking was faster than that of the same generation of the same gender; Ref. no)			
yes	0.93 (0.78–2.44)	1.04 (0.97–1.11)	1.25 (0.82–1.88)
eating breakfast (Ref. not every day)			
eat every day	1.48 (0.90–2.39)	1.01 (0.83–1.22)	1.47 (0.60–3.59)
eating vegetables (Ref. not enough or shortage)			
enough or moderate	1.11 (0.92–1.32)	1.06 (1.00–1.13)	1.08 (0.72–1.62)
eating balanced meals (2 or more times/day; Ref. not every day)			
eat every day	1.20 (0.98–1.48)	0.97 (0.91–1.05)	1.16 (0.74–1.81)
rest due to sleep (Ref. not enough or shortage)			
enough or moderate	1.07 (0.83–1.37)	1.00 (0.91–1.10)	1.38 (0.78–2.45)
connection with neighbors (Ref. weak or unknown)			
strong	1.04 (0.88–1.24)	0.99 (0.93–1.06)	1.19 (0.81–1.77)
having friends (Ref. little or none)			
moderate or a lot	0.98 (0.82–1.17)	1.05 (0.98–1.12)	1.35 (0.91–2.01)

*N* = 500 (community gardener: *n* = 129 and home gardener: *n* = 371). Adjusted for sex, age, family structure, employment status, and gardening style. OR: Odds ratio, CI: Confidence interval, **Bold** means statistically significant (*p* < 0.05).
